# Cyclovirobuxine D Ameliorates Experimental Diabetic Cardiomyopathy by Inhibiting Cardiomyocyte Pyroptosis *via* NLRP3 *in vivo* and *in vitro*

**DOI:** 10.3389/fphar.2022.906548

**Published:** 2022-07-05

**Authors:** Ge Gao, Lingyun Fu, Yini Xu, Ling Tao, Ting Guo, Guanqin Fang, Guangqiong Zhang, Shengquan Wang, Ti Qin, Peng Luo, Xiangchun Shen

**Affiliations:** ^1^ The State Key Laboratory of Functions and Applications of Medicinal Plants, Guizhou Medical University, Guiyang, China; ^2^ The Department of Pharmacology of Materia Medica (The High Efficacy Application of Natural Medicinal Resources Engineering Center of Guizhou Province and The High Educational Key Laboratory of Guizhou Province for Natural Medicinal Pharmacology and Druggability), School of Pharmaceutical Sciences, Guizhou Medical University, Guiyang, China; ^3^ The Key Laboratory of Optimal Utilization of Natural Medicine Resources (The Union Key Laboratory of Guiyang City-Guizhou Medical University), School of Pharmaceutical Sciences, Guizhou Medical University, Guiyang, China; ^4^ The Key Laboratory of Endemic and Ethnic Diseases of Ministry of Education, Guizhou Medical University, Guiyang, China

**Keywords:** cyclovirobuxine D, high glucose, NLRP3 inflammasome, cardiomyocytes, pyroptosis, diabetic cardiomyopathy

## Abstract

Diabetic cardiomyopathy (DCM) is one of the common complications of diabetic patients, which can induce myocardial hypertrophy, cardiac fibrosis, and heart failure. Growing evidence has shown that the occurrence and development of DCM are accompanied by pyroptosis which is an NLRP3-mediated intense inflammatory cell death. Cyclovirobuxine D (CVB-D) has been shown to significantly ameliorate DCM and anti-inflammatory effects associated with cardiomyopathy, but it is unclear whether it has an effect on cardiomyocyte pyroptosis accompanying DCM. Therefore, the purpose of the present study was to explore the ameliorating effect of CVB-D on cardiomyocyte pyroptosis associated with DCM and its molecular regulation mechanism. Type 2 diabetes in C57BL/6 mice was reproduced by the high-fat and high-glucose diet (HFD) combined with low-dose streptozotocin (STZ). The characteristics of DCM were evaluated by cardiac ultrasonography, serum detection, and histopathological staining. The results suggested that CVB-D could significantly alleviate the cardiac pathology of DCM. Then, we explored the mechanism of CVB-D on primary neonatal rat cardiomyocyte (PNRCM) injury with high glucose (HG) *in vitro* to simulate the physiological environment of DCM. Preincubation with CVB-D could significantly increase cell viability, attenuate cytopathological changes and inhibit the expression levels of pyroptosis-related proteins. Further research found that the myocardial improvement effect of CVB-D was related to its inhibition of NLRP3 expression. In conclusion, our data suggest that CVB-D can ameliorate DCM by inhibiting cardiomyocyte pyroptosis *via* NLRP3, providing a novel molecular target for CVB-D clinical application.

## Introduction

Diabetic cardiomyopathy (DCM) is a myocardial microvascular complication of diabetes, characterized by cardiac insufficiency, myocardial interstitial fibrosis, and myocardial hypertrophy, finally resulting in heart failure. Recent evidence has confirmed that DCM is independent of coronary heart disease, hypertension, and other heart diseases (Kaul et al., 2010; Mandavia et al., 2013). It is noteworthy that DCM is the main cause of death in diabetics (Boudina and Abel, 2010; Falcão-Pires and Leite-Moreira, 2012). The pathological mechanisms of DCM include metabolic changes, mitochondrial dysfunction, oxidative stress, inflammation, cell death, extracellular matrix remodeling, etc. (Hu et al., 2017). Pyroptosis is a pro-inflammatory programmed cell death pattern (Kroemer et al., 2009; Coll et al., 2011), which has biochemical and morphological characteristics of necrosis and apoptosis. Unlike apoptosis or necrosis, pyroptosis leads to activating and releasing a large number of inflammatory cytokines (Xu et al., 2014).

Pyroptosis plays a very significant role in the initiation and progression of cardiovascular diseases, so the inhibitors and drugs targeting pyroptosis pathway-related proteins have always been a research hotspot (Jia et al., 2019). More importantly, activation of pro-caspase-1 is the key to initiating pyroptosis. In 2002, Martinon et al. (2002) first identified inflammasome as molecular platforms that trigger activation of cleaved-caspase-1 and then cut pro-IL-1β and pro-IL-18. The inflammasome is a multiprotein complex composed of receptors, connexin ASC, and pro-caspase-1. To date, several types of inflammasome have been identified, including NLRP1, NLRP3, NLRC4, NLRP6, and AIM2 inflammasome. Among these different types of inflammasome, NLRP3 inflammasome has been extensively highlighted in a variety of mammalian cells due to its association with various autoimmune and inflammatory diseases (Zeng et al., 2019). A lot of studies found that hyperglycemia environment–induced overactivation of NLRP3 inflammasome. After the activation of NLRP3, the pyrin domain in NLRP3 binds to the pyrin domain in ASC, which then binds to pro-caspase-1 through card–card interactions. Following pro-caspase-1 is cleaved-caspase-1, an active form, which further promotes the maturation and secretion of IL-18 and IL-1β, and exposes the N-terminal effect domain of GSDMD, a member of the Gasdermins family of proteins. After the formation of membrane pores, pro-inflammatory cytokines and cell contents are released, leading to pyroptosis (Li et al., 2014; Wree et al., 2014; Chen et al., 2016; Xu et al., 2018). It has been reported that the expression of NLRP3, cleaved-caspase-1, and pyroptosis pathway-related proteins are upregulated in the heart of diabetic rats. Inhibition of NLRP3 expression in H9c2 cardiomyocytes can significantly prevent pyroptosis induced by high glucose (Luo et al., 2014). Therefore, several NLRP3 inflammasome inhibitors, such as colchicine, glyburide derivatives, MCC950, INF4E, dapansulide/OLT1177, 16673-34-0, and CY-09, have been identified to prevent NLRP3 oligomerization and interfere with ASC polymerization (Jiang et al., 2017; Toldo and Abbate, 2018; Toldo et al., 2018). In addition to the aforementioned drugs directly targeting NLRP3 inhibition, other molecules that indirectly inhibit NLRP3 expression may also ameliorate cardiovascular diseases. For example, Zhang et al. (2018) found that melatonin rescues endothelial cell pyroptosis by reducing pyroptosis-related protein levels *via* the MEG3/miR-223/NLRP3 signaling axis to ameliorate atherosclerosis. MicroRNA-30c-5p can inhibit NLRP3 inflammation-dependent endothelial cell pyroptosis by downregulating FOXO3 expression in atherosclerosis (Li et al., 2018). Above all, NLRP3, as the initiating signal of pyroptosis, is a potential therapeutic target for tackling cardiovascular diseases.

Cycloxanthine D (CVB-D) is a triterpenoid alkaloid, extracted from Caulis et Ramulus Buxi Sinicae as Chinese medicine, which is widely applied in the prevention and treatment of various cardiovascular diseases such as arrhythmia and heart failure in China. Experiments have confirmed that CVB-D can reduce cardiac hypertrophy in hyperthyroid rats by preventing cardiomyocyte apoptosis and inhibiting the P38 mitogen-activated protein kinase signaling pathway (Wu et al., 2017). It can also alleviate doxorubicin-induced cardiomyopathy by inhibiting oxidative damage and mitochondrial biogenesis disorders (Guo et al., 2015). In addition, previous studies have verified that CVB-D has a significant therapeutic effect on DCM *via* activating the Nrf2-mediated antioxidant response (Jiang et al., 2020). Research studies have reported that CVB-D suppresses lipopolysaccharide-induced inflammatory responses in murine macrophages *in vitro* by blocking the JAK-STAT signaling pathway. The results proved that the anti-inflammatory actions of CVB-D may be related to its cardioprotection (Guo et al., 2014). However, it is still unclear whether CVB-D can improve DCM by inhibiting cardiomyocyte pyroptosis. Therefore, in this study, a pathological model of DCM was established *in vivo* and *in vitro*, with CVB-D treatment. The NLRP3-mediated pyroptosis was evaluated as a signal transduction pathway to investigate the protective effect of CVB-D on DCM and its molecular regulation mechanism. Finally, we found that CVB-D can ameliorate DCM by inhibiting cardiomyocyte pyroptosis *via* NLRP3. Our results add to the evidence for cardiovascular applications of CVB-D.

## Materials and Methods

### Experimental Animals and Groups

Sixty healthy male C57BL/6 mice, 8 weeks old, and weighing 18–24 g were used. The mice were provided by the Animal Experiment Center of Guizhou Medical University, Production License Number: SYXK (Guizhou) 2018-0001. The experiment was approved by the Animal Ethics Committee of Guizhou Medical University (No. 2000904) and followed the ethical standards of animal experiments. The mice were randomly divided into 2 groups with body weight as following: control group (*n* = 12, normal rat maintenance diet) and HFD group (*n* = 48, high-fat and high-glucose diet). The control group mice were fed with a standard diet containing 16% protein, 4% fat, and 60% carbohydrate, while the HFD group mice were fed with HFD containing 18% fat, 20% sucrose, 2% cholesterol, 0.2% cholic acid, and 59.8% normal diet (Jiang et al., 2020). The mice in two groups were free to drink water and chew. After 12 weeks of feeding, serum was collected from the tail vein of mice in each group, and fasting blood glucose (FBG) and insulin (INS) levels were detected to calculate the insulin resistance index. The formula was as follows: HOMA-IR = (FBG × INS)/22.5. Mice in the HFD group with insulin resistance were given a single intraperitoneal injection of streptozotocin (STZ, 30 mg/kg), 10 mg/ml sodium citrate buffer (pH 4.5), and the control group was given an equal volume of sodium citrate buffer. After 4 times of continuous injections of STZ, when the FBG level was higher than 11.1 mM, the mice model of type 2 diabetes was reproduced. Then, the mice in the HFD group were randomly divided into 4 groups as follows: DCM, DCM + CVB-D. L (low dose of CVB-D, 0.5 mg/kg/day), DCM + CVB-D. H (high dose of CVB-D, 1 mg/kg/day), and DCM + Met (metformin, 250 mg/kg/day). The control and DCM groups were given saline for the next 2 months. CHO (Cholesterol, Redu Life Sciences Co., Ltd., China), TG (Triglycerides, Redu Life Sciences Co., Ltd., China), and LDH (Lactate dehydrogenase, Nanjing Jiancheng Institute of Biological Engineering, China) contents in serum were detected after the experiment.

### Cell Culture and Treatment

After extraction, isolation, purification, culture, and identification of PNRCMs (primary neonatal rat cardiomyocytes), the protective effects of CVB-D on HG (high glucose)-induced PNRCM injury were investigated. The experimental groups were as follows: Control (25 mM glucose Dulbecco’s modified eagle medium, 25 mM glucose DMEM), HG (40 mM glucose); HG + CVB-D. L (0.1 μM), HG + CVB-D. H (1 μM), and HG + Met (0.5 mM), PNRCMs were preincubated with CVB-D and Met for 1 h and then treated with HG for 24 h. Preliminary basic studies have proved that CVB-D has a significant inhibitory effect on NLRP3-mediated cardiomyocyte pyroptosis. In order to further clarify the molecular mechanism of CVB-D on inhibiting PNRCM pyroptosis, NLRP3 inhibitor (MCC950, MedChemExpress, United States) and agonist (BMS-986299, MedChemExpress, United States) were added. The experimental design was as follows: Control (25 mM glucose DMEM), HG (40 mM glucose), HG + CVB-D (1 μM), HG + MCC950 (1 μM) or BMS-986299 (1 μM), and HG + CVB-D + MCC950 or BMS-986299. PNRCMs were pretreated with MCC950 or BMS-986299 for 1 h, then CVB-D was added for 1 h, and finally co-incubated with HG for 24 h.

### Ultrasonography

First, the mice were anesthetized by ether inhalation. The limbs of the mice were fixed after anesthesia, and then the hair on the chest was removed with hair removal cream. The coupling agent was evenly applied after the skin was exposed. The EF (left ventricular ejection fraction), FS (fractional shortening), IVSd (interventricular septal thickness at diastole), LVPWd (left ventricular posterior wall dimensions), and LVIDd (left ventricular internal diameter at end-diastole) of mice were recorded with the M-mode of small-animal ultrasonography (Feiyinuo Technology Co., Ltd., China).

### Hematoxylin-Eosin Staining

The heart tissues were fixed in 4% paraformaldehyde for more than 24 h, and then were dehydrated and embedded. The repaired wax blocks were placed in a paraffin slicer for sections and the thickness was 3 μm. After the paraffin sections were dewaxed, the following staining steps were performed respectively: hematoxylin staining, eosin staining, dehydration, and sealing. Finally, microscopic examination, image acquisition, and analysis (Nikon, Japan) were carried out by independent pathological workers.

### Masson Staining

After the paraffin sections were dewaxed to water, we stained successively with potassium dichromate, hematoxylin, lichun red acid fuchsin, phosphomolybdic acid, and aniline blue. The sections were sealed after differentiation. Images data were analyzed using a microscope (Nikon, Japan).

### TUNEL Staining

The sections were stained according to the instructions of the terminal deoxynucleotidyl transferase–mediated dUTP-biotin nick end labeling (TUNEL) staining kits (Roche, Switzerland). DAPI was used for nuclear staining, and the sections were sealed. They were observed using a fluorescence microscope, and images were collected (Nikon, Japan).

### Immunohistochemistry

After paraffin sections’ dewaxing, the following steps were conducted: antigen repair, blocking endogenous peroxidase, serum sealing, adding primary and secondary antibodies, DAB coloration, nucleus staining, dehydration, sealing, microscopic observation, and capturing images (CIC, United States).

### MTT Assay

The PNRCMs were seeded into 96-well plates and cultured for 72 h. After 24 h of treatment, 20 μl 5 mg·ml^−1^ MTT was added (Beijing Solaibao Technology Co., Ltd., China) to each well and incubated for 4–6 h under dark conditions. Then the supernatant of the cardiomyocytes culture was discarded. After 150 μl DMSO was added to each well, the plate was placed on the shaker until all the crystals at the bottom dissolved. Finally, the absorbance at 490 nm wavelength was measured (Thermo scientific, United States).

### Giemsa Staining

PNRCMs were fixed with 4% paraformaldehyde for 20 min, and then stained with staining solution A and B in turn according to the instructions (Beijing Solaibao Technology Co., Ltd., China). After the sediment in the staining solution was washed out, cell morphology was observed under an inverted microscope, and images were taken (Chongqing Photoelectric Instrument Co., Ltd. China).

### Immunofluorescence

After the cells were fixed, permeated, and sealed with goat serum, the primary and secondary antibodies were dropped and incubated. The nucleus was stained with DAPI and photographed with a fluorescence microscope (Leica, Germany). The fluorescence values of each group were detected by ImageJ software.

### Calcein-AM/PI Staining

The cells were rinsed with phosphate buffer saline (PBS) two times and incubated with Calcein-AM and PI (Shanghai Bebo Biological Technology Co., Ltd., China) at room temperature for 40 min without light, respectively. The cells were rinsed with PBS again twice and observed using the fluorescence microscope (Leica, Germany).

### ELISA

IL-18 and IL-1β were determined in mice serum and supernatant of cardiomyocytes culture according to the kit instructions provided by Shanghai Fanyin Biotechnology Co., Ltd. (China).

### Virus Transfection

To determine the role of NLRP3 expression in CVB-D inhibition of cardiomyocyte pyroptosis, we designed transfecting cardiomyocytes with NLRP3-shRNA lentivirus. NLRP3-shRNA and shRNA-NC were from Hunan Fenghui Biotechnology Co., Ltd. (China). For NLRP3-shRNA sequences were: GATCCGGCAGGTTCTACTCCATCAAAGTTCAAGAGActttgatggagtagaacctgcTTTTTG.Virus titer: 1.1 × 10^8^ (TU/mL).

### Western Blotting

Mice heart tissues and cardiomyocytes were lysed with the RIPA buffer (Beijing Solaibao Technology Co., Ltd., China) to obtain total proteins. The concentration of extracted proteins was determined by using a BCA protein concentration analysis kit (Beijing Solaibao Technology Co., Ltd., China). The proteins with equal mass were separated into EP tubes, and then they were separated by SDS-PAGE and transferred to the PVDF membrane. After blocking with 5% skim milk at room temperature for 1.5 h, the membranes were incubated with the following primary antibodies at 4°C overnight: NLRP3 (Proteintech, China), pro-caspase-1 (Changzhou Xiangtai Biotechnology Co., Ltd., China), cleaved-caspase-1 (Suzhou Ruiying Biotechnology Co., Ltd, China), IL-18 (Proteintech, China), GSDMD &GSDMD-N (Changzhou Xiangtai Biotechnology Co., Ltd., China), IL-1β (Changzhou Xiangtai Biotechnology Co., Ltd., China), and GAPDH (Proteintech, China). The next day, the membranes were incubated with the corresponding secondary antibody at room temperature for 1.5 h. The ECL buffer (New Sai Mei Biotechnology Co., Ltd., China) was added to the membrane. The protein bands were scanned by Bio-RAD ChemiDoc XRS + imaging system (United States). Image Lab 4.0 software was used for stripe gray value statistical analysis.

### Statistical Analysis

The experimental data were analyzed and processed by GraphPad Prism software, and the results were expressed as the mean ± SEM. *p* < 0.05 was considered statistically significant.

## Results

### CVB-D Improves Cardiac Dysfunction in DCM Mice

**FIGURE 1:**
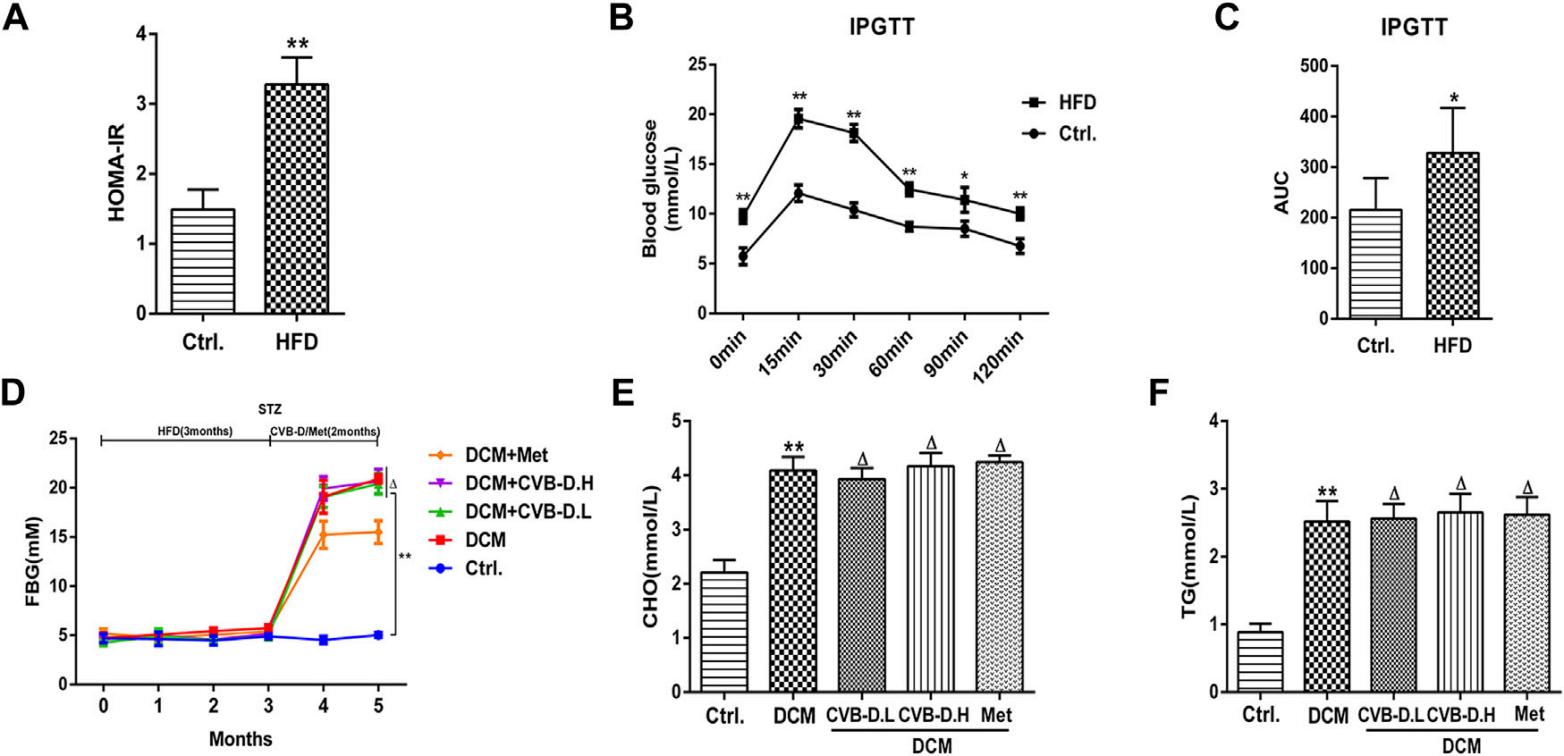
HFD (high-fat and high-glucose diet) combined with STZ (streptozotocin) induces type 2 diabetes model in mice. **(A)** Insulin resistance index was calculated in mice by detecting FBG (fasting blood glucose) and INS (insulin). **(B)** After intraperitoneal injection of glucose in fasting condition, the blood glucose of mice at different time points was measured. **(C)** Histogram of AUC (area under the curve) of blood glucose at different time points in mice. **(D)** FBG of mice during modeling and treatment. **(E,F)** Effects of CVB-D on CHO (cholesterol) and TG (triglycerides) contents in serum of DCM (diabetic cardiomyopathy) mice. The data are presented as the mean ± SEM (*n* = 6), *p < 0.05, **p < 0.01 versus the control group; ^Δ^ *p* > 0.05 versus the model group.

**FIGURE 2:**
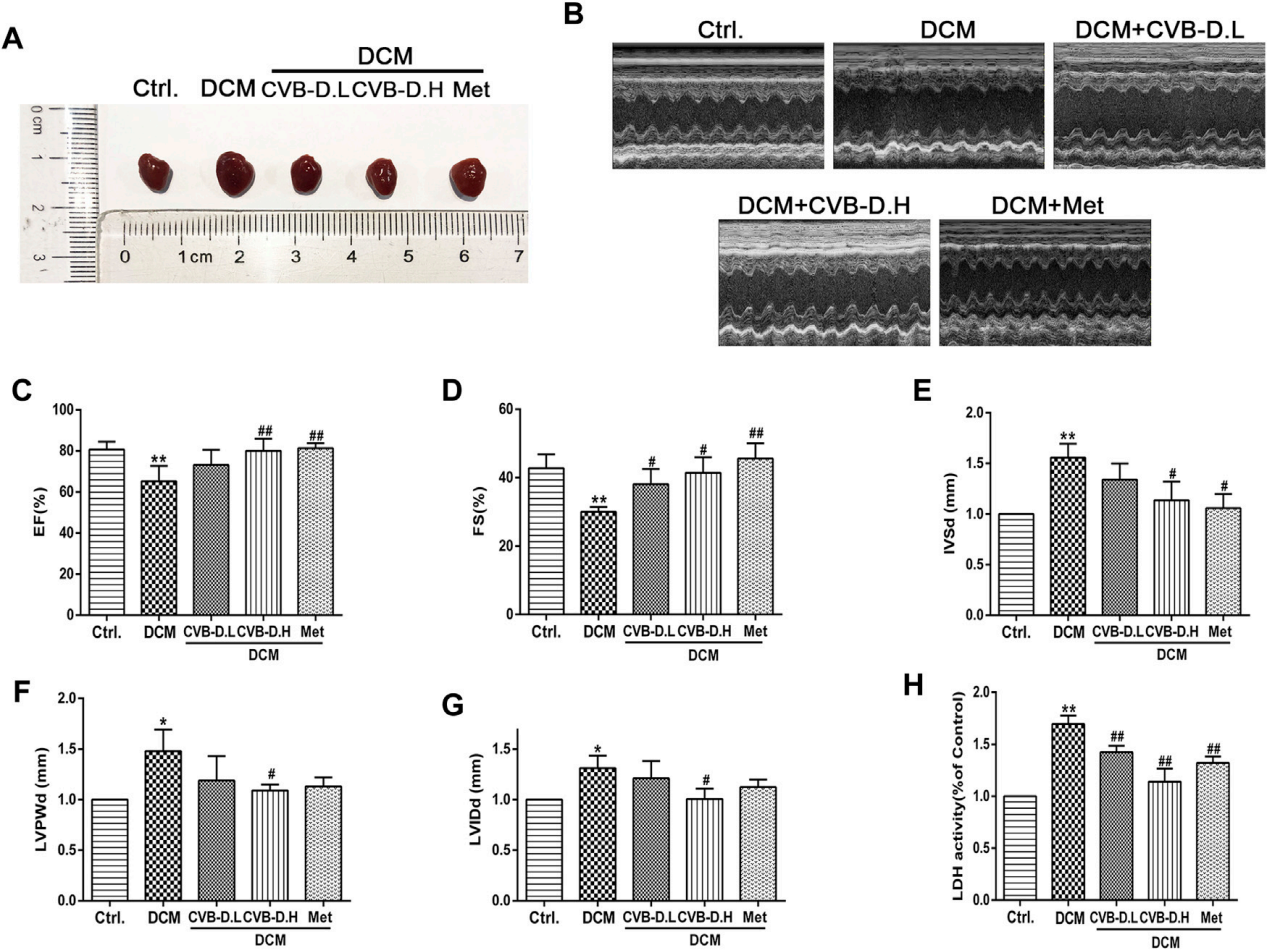
CVB-D improves cardiac dysfunction in DCM mice. **(A)** Effects of CVB-D on cardiac hypertrophy in DCM mice. **(B)** Cardiac function of mice was measured by cardiac ultrasound. **(C–G)** The effect of CVB-D on EF (left ventricular ejection fraction), FS (fractional shortening), IVSd (interventricular septal thickness at diastole), LVPWd (left ventricular posterior wall dimensions), LVIDd (left ventricular internal diameter at end-diastole) of DCM mice hearts. **(H)** The content of LDH (lactate dehydrogenase) in serum of mice was detected. The data are presented as the mean ± SEM (*n* = 6), *p < 0.05, **p < 0.01 versus the control group; ^#^ *p* < 0.05, ^##^ *p* < 0.01 versus the model group.

The type 2 diabetes model was reproduced by HFD and intraperitoneal injection of STZ in mice. In Figure 1A, the model mice showed significant insulin resistance after 12 weeks of HFD compared with the control group. Results of IPGTT (intraperitoneal glucose tolerance test) showed that the blood glucose of mice in the HFD group was significantly higher than the control group at 0, 15, 30, 60, 90, and 120 min after intraperitoneal glucose injection (Figure 1B), and the AUC (area under the curve) of blood glucose was also larger than the control group (Figure 1C). In addition, the FBG, CHO, and TG in mice serum of the model group were significantly increased compared to those in the control group; however, CVB-D did not improve these three indicators, suggesting that CVB-D could not ameliorate metabolic disorder in type 2 diabetes mice (Figures 1D–F). Compared with the control group, the cardiac hypertrophy of mice in the model group was alleviated by CVB-D treatment for 2 months (Figure 2A). Cardiac ultrasonography was widely applied to monitor the cardiac function of mice, and our data indicated that the cardiac systolic and diastolic function deteriorated in EF, FS, IVSd, LVPWd, and LVIDd of DCM mice. After CVB-D treatment, these values were reversed and the cardiac function of mice was improved (Figures 2B–G). In addition, CVB-D was found to significantly reduce serum LDH content in DCM mice (Figure 2H). Furthermore, compared with the control group, the serum levels of IL-18 and IL-1β in DCM mice were increased, and CVB-D treatment could inhibit the serum levels of these two pro-inflammatory cytokines (Figures 4D,E). Taken together, DCM can be induced by type 2 diabetes, which leads to cardiac hypertrophy, cardiac systolic and diastolic dysfunction, and the release of pro-inflammatory cytokines. All of the harmful changes can be reversed by CVB-D, and these improvement effects do not affect the metabolic disorders of DCM mice.

### CVB-D Prevents Cardiomyocyte Pyroptosis in DCM Mice

**FIGURE 3:**
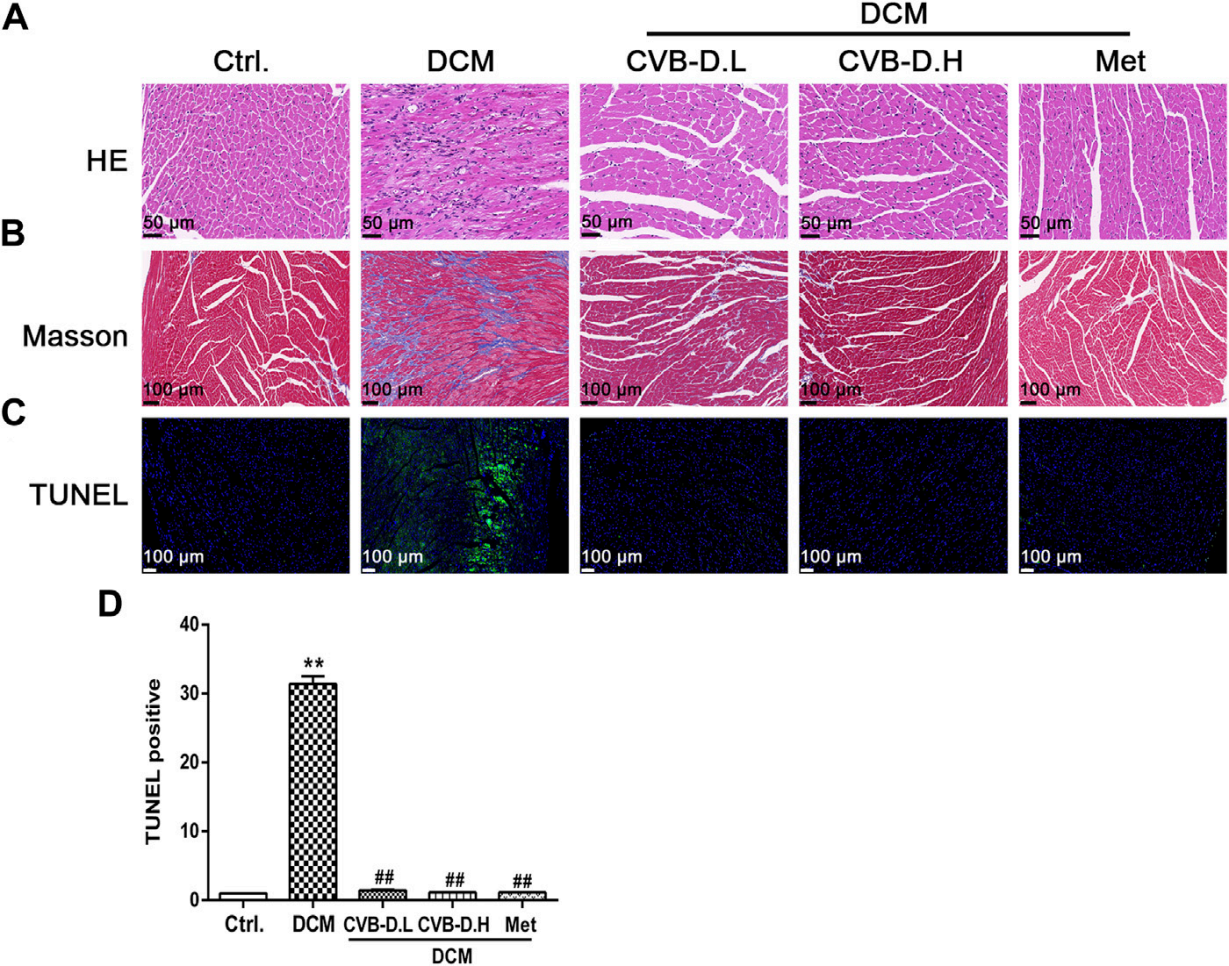
CVB-D ameliorates the cardiac physiology changes in DCM mice. **(A)** HE (Hematoxylin-Eosin) staining of mice heart tissues. **(B)** Masson staining was performed in mice heart tissues. **(C,D)** Influence of CVB-D on TUNEL positive cell number. The data are presented as the mean ± SEM (*n* = 6), *p < 0.05, **p < 0.01 versus the control group; ^#^ *p* < 0.05, ^##^ *p* < 0.01 versus the model group.

Hematoxylin–eosin (HE) staining showed that DCM mice had cardiomyocyte arrangement disorder, cell hypertrophy, and obvious inflammatory cell infiltration; however, CVB-D significantly ameliorated these pathological changes induced by DCM (Figure 3A). Masson staining, as a pathological check for fibrosis, indicates that DCM could result in severe myocardial fibrosis in mice, and CVB-D treatment significantly reduced collagen deposition (Figure 3B).

**FIGURE 4:**
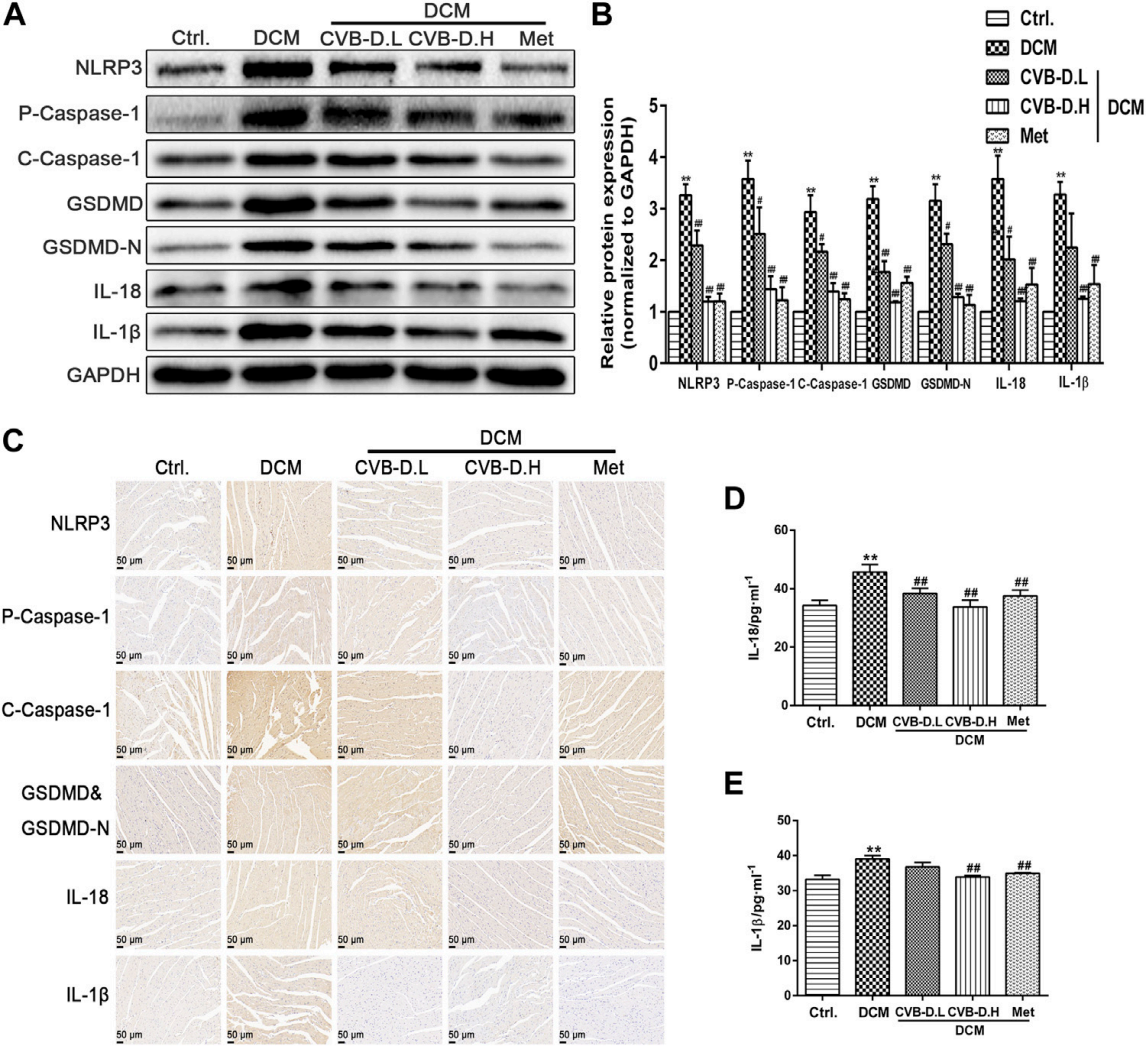
CVB-D inhibites the expression of pyroptosis-related proteins in the heart tissue of DCM mice. **(A,B)** Determination of NLRP3 and pyroptosis pathway-related proteins expression in heart tissues of DCM mice by western blotting, and all proteins expression were normalized to GAPDH. **(C)** The expressions of NLRP3, P-Caspase-1 (Pro-Caspase-1), C-Caspase-1 (Cleaved-Caspase-1), GSDMD&GSDMD-N, IL-18 and IL-1β were observed by immunohistochemistry. **(D,E)** Effects of CVB-D on the contents of IL-18 and IL-1β in mice serum. The data are presented as the mean ± SEM (*n* = 6), *p < 0.05, **p < 0.01 versus the control group; ^#^ *p* < 0.05, ^##^ *p* < 0.01 versus the model group.

Pyroptosis exhibits some of the morphological characteristics of both apoptosis and necrosis, one of which is DNA fragmentation, so positive results were found in the TUNEL test (Luo et al., 2014). Experimental results suggested that nuclear DNA damage in DCM mice was more serious than in the control group, while CVB-D reversed the phenomenon of myocardial nuclear DNA fragmentation (Figures 3C,D). NLRP3-mediated cardiomyocyte pyroptosis is critical for the initiation and development of DCM, so we measured the protein expression levels of NLRP3, p-caspase-1 (pro-caspase-1), c-caspase-1 (cleaved-caspase-1), GSDMD, GSDMD-N, IL-18, and IL-1β in mice heart tissue by Western blot. Experiment results showed that the expression levels of NLRP3 and pyroptosis pathway-related proteins were significantly increased in the DCM group, and CVB-D could attenuate the expression of these proteins (Figures 4A,B). Immunohistochemical staining results of heart tissues were consistent with Western blot results (Figure 4C). Above all, the present results indicate that CVB-D can significantly improve the cardiac pathological changes in DCM mice, which may be related to the inhibition of NLRP3-mediated cardiomyocyte pyroptosis.

### CVB-D Improves the PNRCM Injury Induced by HG

**FIGURE 5:**
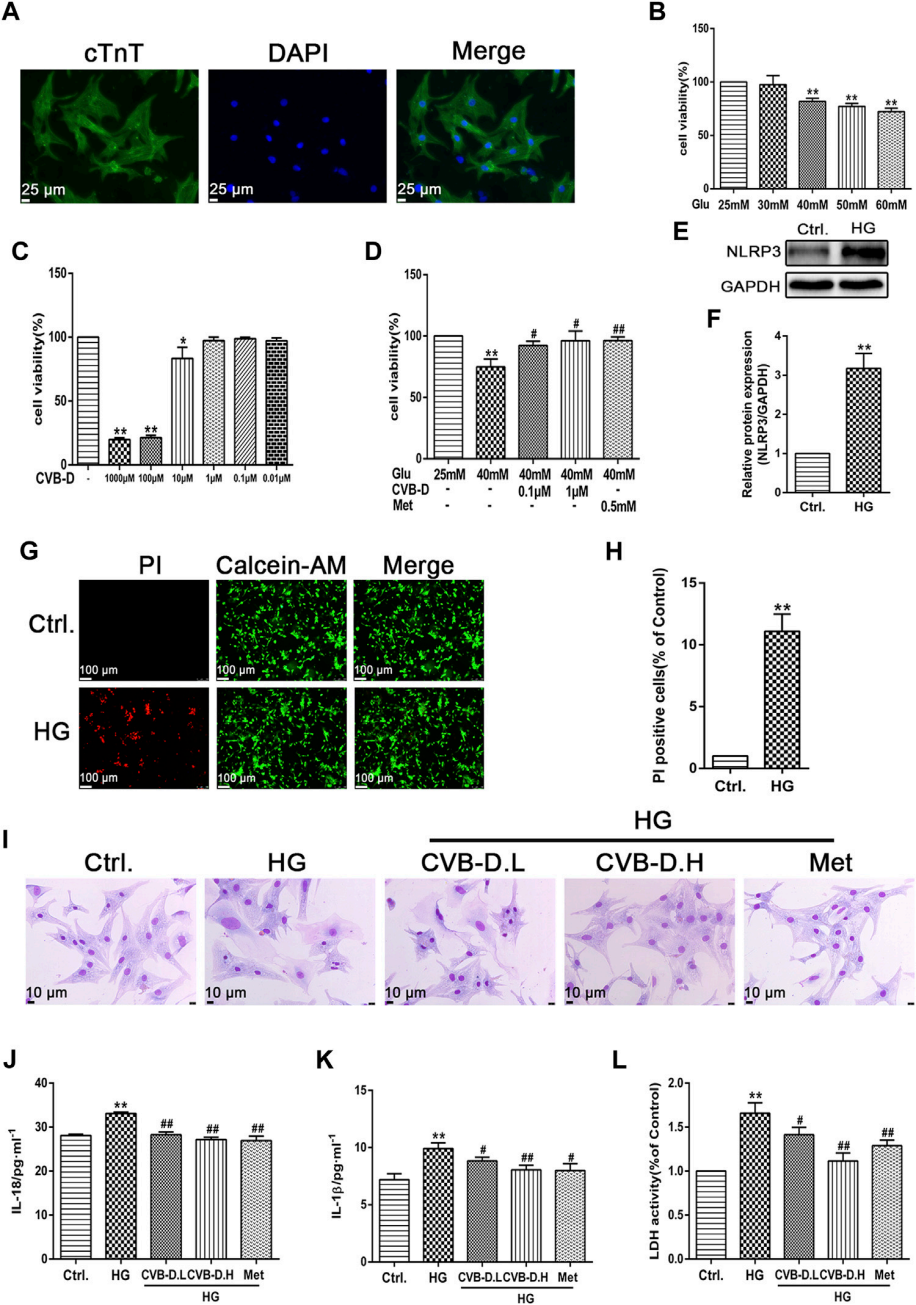
CVB-D improves the PNRCMs (primary neonatal rat cardiomyocytes) injury induced by HG (high glucose). **(A)** Immunofluorescence was used to identify the purity of PNRCMs. **(B–D)** The effect of CVB-D on the decreased viability of PNRCMs induced by HG was detected by MTT assay. **(E,F)** Western blotting was used to determine the effect of HG on NLRP3 expression in PNRCMs. **(G,H)** Calcein-AM/PI staining was used to observe cardiomyocyte pyroptosis caused by HG. **(I)** Effects of CVB-D on pathological changes of PNRCMs induced by HG were observed by Giemsa staining. **(J–L)** Influence of CVB-D on the contents of IL-18, IL-1β and LDH in the supernatant of PNRCMs culture medium were detected by ELISA and LDH kits. The data are presented as the mean ± SEM (*n* = 3), *p < 0.05, **p < 0.01 versus the control group; ^#^ *p* < 0.05, ^##^ *p* < 0.01 versus the model group.

In order to simulate the physiological environment of DCM, PNRCMs were exposed to 40 mM HG. First, PNRCMs were extracted by the trypsin digestion method and purified by the differential speed sticking method and then cultured in DMEM medium *in vitro*. The PNRCMs were identified by immunofluorescence staining with cardiomyocyte-specific protein–cardiac troponin T (cTnT) (Figure 5A). MTT results suggested that 25 mM glucose had no effect on the viability of PNRCMs, while 40 mM HG significantly reduced the viability of PNRCMs (Figure 5B). The cytotoxicity test results of CVB-D showed that the concentration of CVB-D had no toxicity on cell viability less than 1 μM (Figure 5C). 0.1 μM or 1 μM CVB-D could reverse the decreased viability of PNRCMs induced by 40 mM HG (Figure 5D). Western blot analysis indicated that HG upregulated the expression level of NLRP3 (Figures 5E,F) and induced PNRCMs pyroptosis, resulting in a significantly higher number of PI stained positive cells than the control group (Figures 5G,H).

Exposure to HG resulted in hypertrophy of PNRCMs, unclear cell membranes, spherical cells with increased oxygen consumption, and nucleus moving to the cell center by Giemsa staining. Preincubation with CVB-D could significantly ameliorate cellular morphology (Figure 5I). Further, IL-18 and IL-1β contents, and LDH release were determined by commercial kits in the cell culture supernatant. Experimental results showed that HG induced the secretion of large amounts of IL-18 and IL-1β into the culture medium, and LDH released leakage as well. The CVB-D could inhibit the increase of IL-18 and IL-1β, and release leakage of LDH in the medium (Figure 5J–L). To sum up, CVB-D could alleviate the damage and pathological changes of PNRCMs induced by HG, thus playing a protective role in the myocardium.

### CVB-D Inhibits PNRCMs Pyroptosis Induced by HG

**FIGURE 6:**
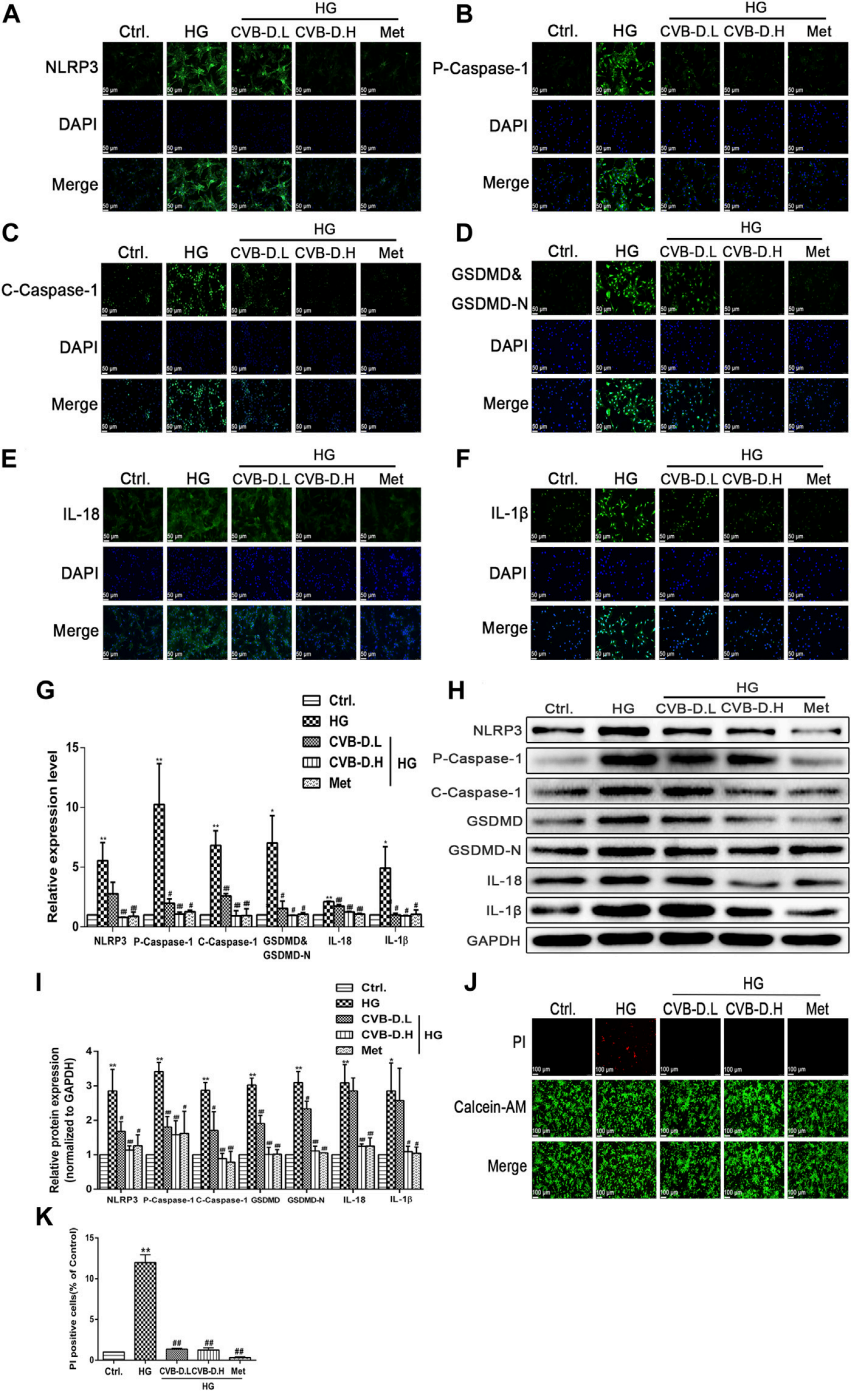
CVB-D inhibits PNRCMs pyroptosis induced by HG. **(A–F)** The effect of CVB-D on the expression of pyroptosis pathway proteins was observed by immunofluorescence. **(G)** The fluorescence values of each group were counted by ImageJ software. **(H,I)** Western blotting was used to determine the expression level of pyroptosis related proteins after CVB-D treatment. **(J,K)** Pyroptosis and living cells were differentiated by Calcein-AM/PI staining after CVB-D treatment. The data are presented as the mean ± SEM (*n* = 3), *p < 0.05, **p < 0.01 versus the control group; ^#^ *p* < 0.05, ^##^ *p* < 0.01 versus the model group.

In order to further investigate the inhibitory effect of CVB-D on PNRCMs pyroptosis induced by HG, the expression levels of NLRP3, p-caspase-1 (pro-caspase-1), c-caspase-1 (cleaved-caspase-1), GSDMD, GSDMD-N, IL-18, and IL-1β were assayed using the immunofluorescence method. The results showed that NLRP3 was greatly activated under high-glucose stimulation, and the activated NLRP3 induced pro-caspase-1 activation, as well as downstream GSDMD, GSDMD-N, IL-18, and IL-1β maturation. CVB-D inhibited the expression of these relevant proteins, that is, preventing PNRCM pyroptosis (Figures 6A–G). After extracellular proteins were extracted, the results of Western blot were consistent with the immunofluorescence staining results (Figures 6H,I). We further observed the DNA breakage and cell membrane damage of cardiomyocytes by PI staining and found that CVB-D could significantly reduce the number of PI-stained positive cells. So, we thought CVB-D reversed PNRCMs pyroptosis induced by HG (Figures 6J,K). It was found that CVB-D improves the PNRCM pyroptosis caused by HG.

### CVB-D Ameliorates HG-Induced PNRCM Injury by Inhibiting Cardiomyocyte Pyroptosis *via* NLRP3

**FIGURE 7:**
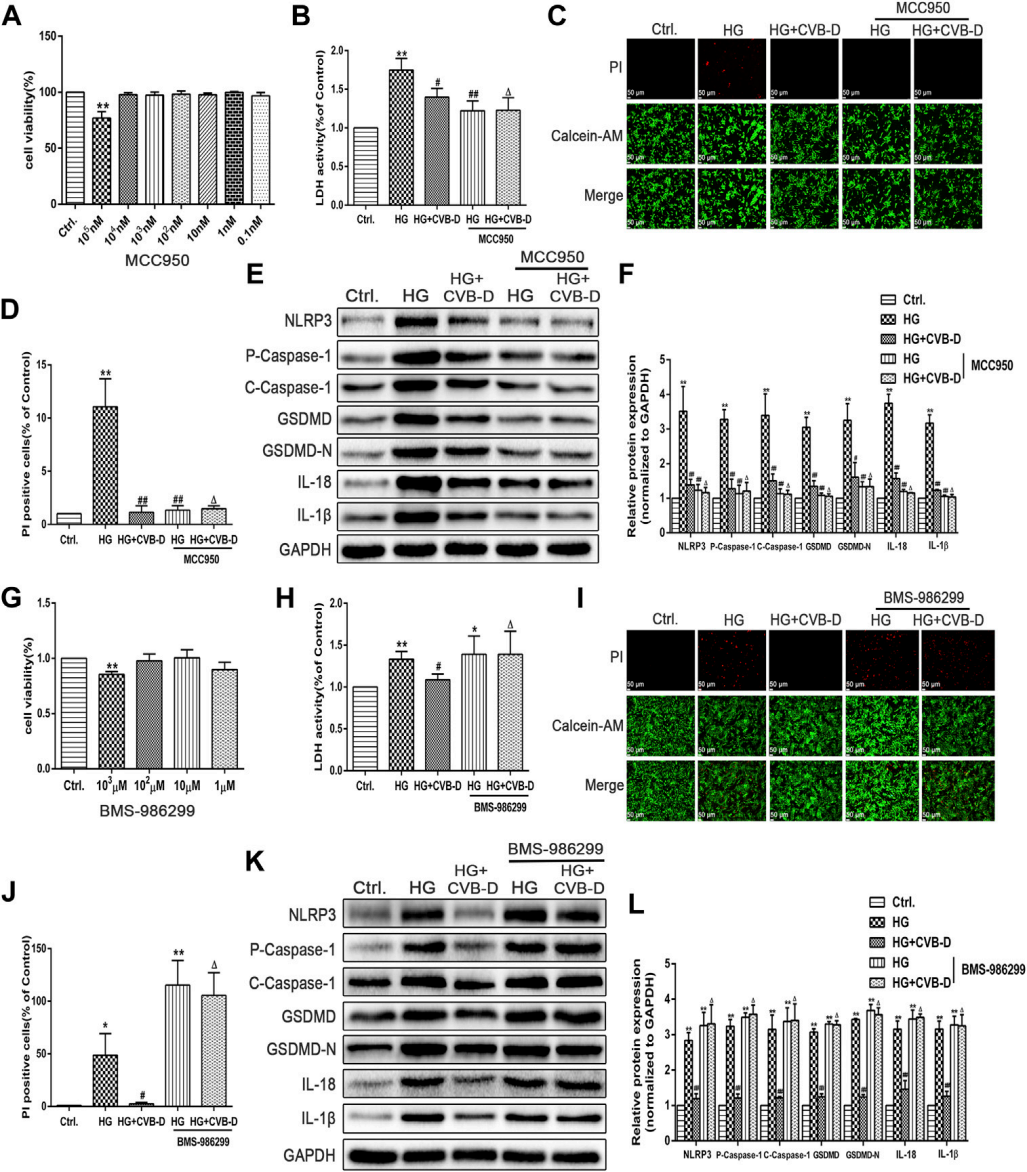
CVB-D ameliorates HG-induced PNRCMs injury by inhibiting cardiomyocyte pyroptosis via NLRP3. **(A)** Cytotoxicity of MCC950 was detected by MTT assay. **(B)** After pretreatment with MCC950, LDH content in cell culture supernatant was detected. **(C,D)** The effect of MCC950 on cardiomyocyte pyroptosis was judged by the number of PI staining positive cells. **(E,F)** The expression level of pyroptosis related proteins was detected by western blotting with MCC950 pretreatment. The data are presented as the mean ± SEM (*n* = 3), *p < 0.05, **p < 0.01 versus the control group; #*p* < 0.05, ##*p* < 0.01 versus the model group; Δ*p* > 0.05 versus the HG+MCC950 group. **(G)** Cytotoxicity of BMS-986299 was detected by MTT assay. **(H)** LDH content in cell culture supernatant was detected after pretreatment with BMS-986299. **(I,J)** The effect of BMS-986299 on cardiomyocyte pyroptosis was observed by PI staining. **(K,L)** Western blotting was used to detected proteins expression in pyroptosis pathway with BMS-986299 pretreatment. The data are presented as the mean ± SEM (*n* = 3), *p < 0.05, **p < 0.01 versus the control group; ^#^ *p* < 0.05, ^##^ *p* < 0.01 versus the model group; ^Δ^ *p* > 0.05 versus the HG+BMS-986299 group.

The pharmacological NLRP3 inhibitor (MCC950) and agonist (BMS-986299) were used to investigate the role of NLRP3 in CVB-D inhibition of pyroptosis in HG-induced PNRCMs. Cytotoxicity of MCC950 and BMS-986299 were determined by MTT assay (Figures 7A,G). Then, we detected LDH releasing leakage in the cell culture supernatant, NLRP3, and pyroptosis pathway-related proteins expression levels. The results suggested that MCC950 inhibited the expression of NLRP3 and downregulated the expression of pyroptosis-related proteins. Thus, MCC950 inhibited cardiomyocyte pyroptosis and alleviated LDH leakage caused by HG. For the LDH content in the medium and Western blot, the results showed that there was no statistical difference compared to HG + MCC950 with HG + CVB-D + MCC950 groups (Figures 7B,E,F). The results of the number of positive cells stained by PI were the same as the Western blot results (Figures 7C,D). In addition, the results of calcein-AM/PI staining suggested that the inhibition effect of CVB-D on cardiomyocyte pyroptosis was abrogated by BMS-986299, which also increased CVB-D–induced downregulation of LDH content in the medium (Figures 7H–J). The pyroptosis pathway-related protein expression was determined by Western blot. Compared with the HG + BMS-986299 group, HG + CVB-D + BMS-986299 could not further affect the expression of pyroptosis-related proteins (Figures 7K,L).

**FIGURE 8:**
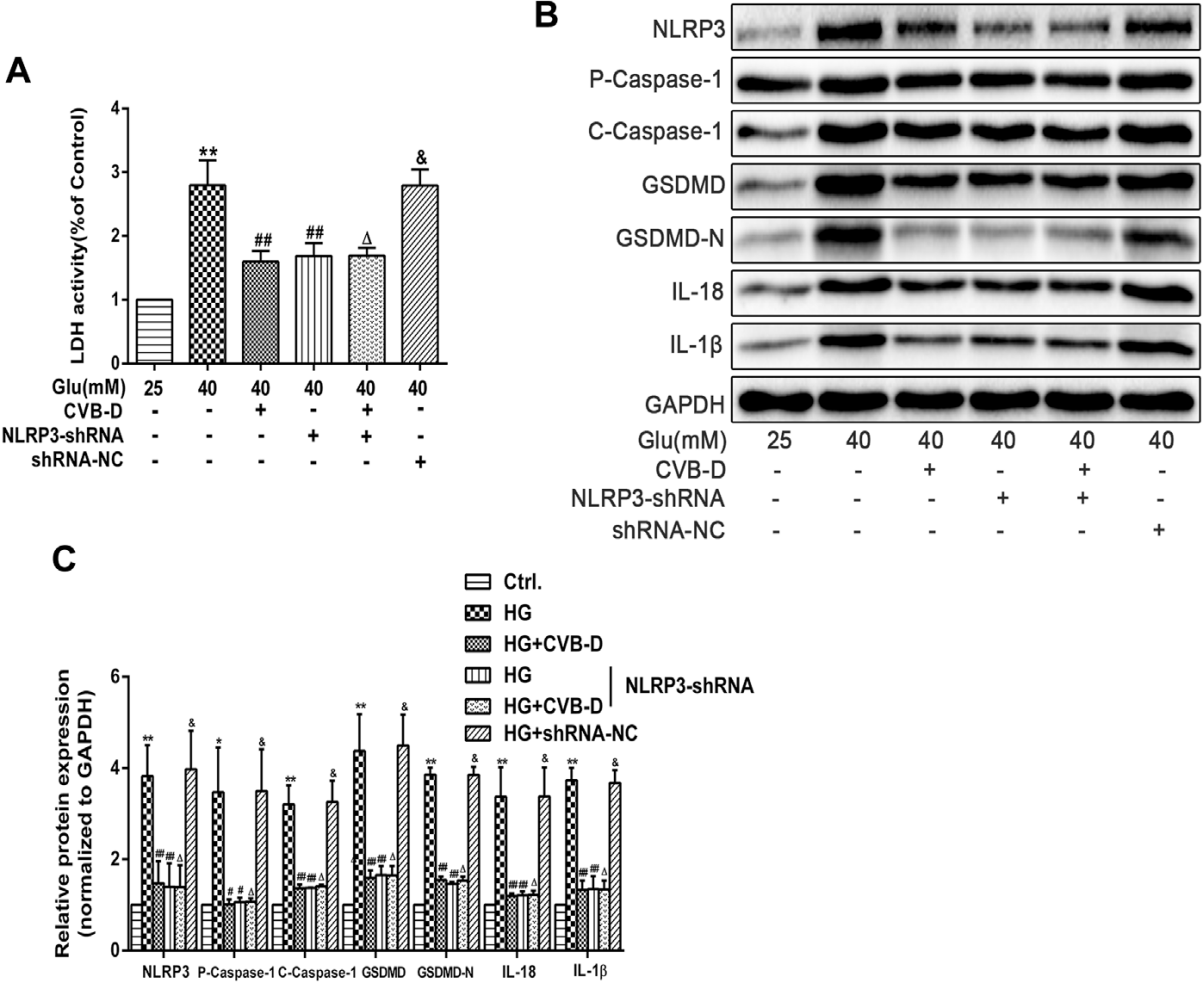
NLRP3-shRNA verifies the regulation mechanism of CVB-D inhibition pyroptosis induced by HG in PNRCMs. **(A)** LDH release in cell culture supernatant was detected after NLRP3-shRNA lentivirus-infected. **(B,C)** Western blotting was used to detected proteins expression in pyroptosis pathway after NLRP3-shRNA lentivirus infection with PNRCMs. The data are presented as the mean ± SEM (*n* = 3), *p < 0.05, **p < 0.01 versus the control group; ^#^ *p* < 0.05, ^##^ *p* < 0.01 versus the model group; ^Δ^ *p* > 0.05 versus the HG+NLRP3-shRNA group; ^&^ *p* > 0.05 versus the model group.

To further clarify the role of NLRP3 in CVB-D inhibition PNRCMs pyroptosis, we transfected NLRP3-shRNA lentivirus into PNRCMs. The results showed that NLRP3-shRNA and CVB-D significantly reduced the expression of NLRP3 and pyroptosis pathway-related proteins. Consistently, LDH leakage results and protein expression results showed no significant difference between the HG + NLRP3-shRNA group and the HG + NLRP3-shRNA + CVB-D group (Figures 8A–C). Our results consistently showed that CVB-D ameliorated HG-induced PNRCMs pyroptosis, at least in part, *via* NLRP3.

## Discussion

Cardiovascular complications from diabetes have been the leading cause of morbidity and mortality worldwide. DCM is a descriptive pathology that can be defined as changes in the myocardial structure and function independent of other conventional cardiac risk factors, including hypertension, coronary atherosclerotic heart disease (CAD), and significant heart valve disease in diabetic patients (Bell and Goncalves, 2019). DCM has no clinical symptoms in the early stages of its progression (Jia et al., 2016). In the first stage, it mainly presents as various forms of cardiometabolic abnormalities, which may be associated with increased myocardial fibrosis and stiffness. Those harmful changes further lead to atrioventricular filling dysfunction and increased left ventricular end-diastolic pressure (Westermeier et al., 2016). The second stage is the evolution of DCM to heart failure, followed by cardiac remodeling, left ventricular hypertrophy, myocardial interstitial fibrosis, and diastolic dysfunction, which further leads to heart failure with reduced ejection fraction (Jia et al., 2016). Due to the complexity of the pathogenesis and clinical characteristics of DCM, the clinical treatment of DCM is facing a great challenge.

Although extensive research has been carried out in the past 10 years, there is still no convincing method for the treatment of DCM. In 2008, the United States Food and Drug Administration (USFDA) also proposed that for diabetes-induced cardiovascular complications, only controlling blood glucose is not enough (Kant et al., 2019), so it is necessary to further explore the pathological basis of DCM. According to literature reports, many harmful factors, such as oxidative stress, inflammation, cardiomyocyte death, interstitial fibrosis of cardiac tissue, and cardiac stiffness, lead to diastolic and systolic dysfunction. These lesions are the initiation and progression of DCM, and ultimately result in heart failure (Palmieri et al., 2001; Levelt et al., 2016). NLRP3, the most widely studied type of inflammasome, is involved in a variety of physiological processes *in vivo* (Zeng et al., 2019). In addition, accumulating evidence has shown that NLRP3 overexpression can be induced in a hyperglycemic environment, leading to pyroptosis, a strong pro-inflammatory cell death mode (Kroemer et al., 2009; Coll et al., 2011). Recently, it is well known that pyroptosis accelerates the deterioration of DCM (Luo et al., 2014). Above all, based on the complexity of the pathological basis of DCM, combined with the laboratory preliminary studies, we have confirmed that CVB-D, an important pharmacodynamic component of Chinese medicine Caulis et Ramulus Buxi Sinicae, could improve DCM by activating Nrf2-mediated antioxidant reaction (Jiang et al., 2020). Therefore, we further explored the molecular regulatory mechanism of CVB-D on the improvement of DCM by taking the NLRP3-mediated cardiomyocyte pyroptosis as the research route.

We successfully reproduced the type 2 diabetes model *in vivo* through a high-fat and high-glucose diet and low-dose STZ. The experimental results suggested that compared with the control group, DCM mice showed significant cardiac hypertrophy and cardiac systolic and diastolic dysfunction. The mice in the model group also showed many harmful changes, such as increased levels of serum inflammatory cytokines IL-18 and IL-1β, which were consistent with the clinical characteristics of DCM (Jia et al., 2016; Westermeier et al., 2016). In addition, we observed the pathological changes in the heart tissue in mice and found obvious inflammatory cell infiltration, myocardial fibrosis, and DNA breakage. These results suggested that inflammation plays a key role in the progression of DCM. CVB-D alleviated the aforementioned deterioration, indicating that the improvement of DCM by CVB-D was related to the inhibition of inflammatory response.

**FIGURE 9:**
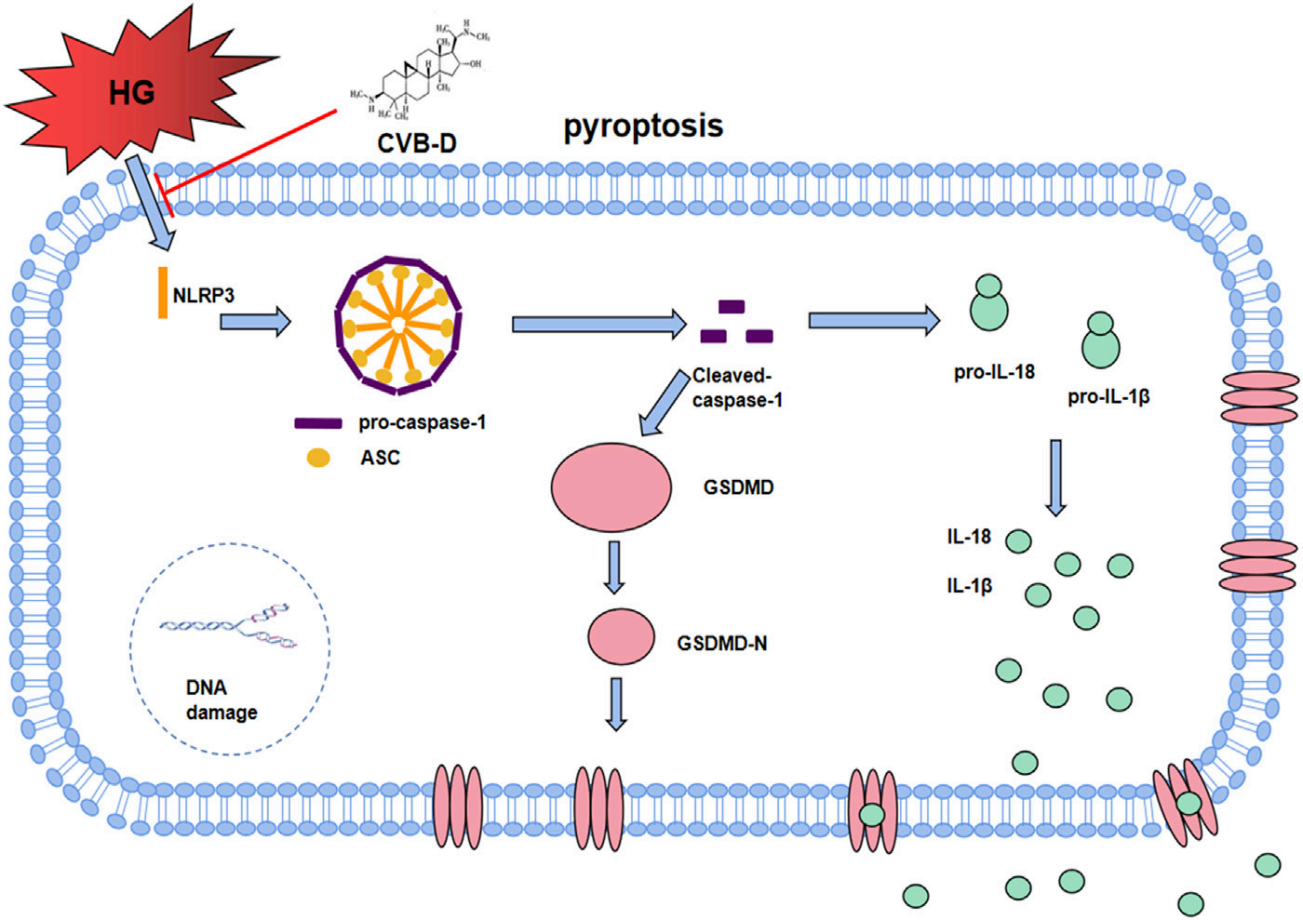
The molecular regulation mechanism of CVB-D improving DCM. After HG induced NLRP3 overactivation, it assembles with ASC and pro-caspase-1 to form inflammasome. After Cleaved-caspase-1 is activated, on the one hand, it activates GSDMD-N to cause pore formation on cell membrane. On the other hand, Cleaved-caspase-1 promotes the maturation and secretion of IL-18 and IL-1β, finally leads to cardiomyocyte pyroptosis, accelerating the progress of DCM. CVB-D significantly ameliorates DCM, which may be associated with the inhibition of NLRP3-mediated cardiomyocyte pyroptosis.

In the NLRs family, NLRP3 has been identified as a key node-like receptor family member that can recognize microbial and non-microbial danger signals and trigger aseptic inflammatory responses in various disease conditions (Yang et al., 2018; Jin et al., 2021). Recognition signals are transduced to the inflammasome adapter ASC (apoptosis-associated speck-like protein containing a CARD) to further activate pro-caspase-1, followed by IL-18/1β release and GSDMD cleavage to induce pyroptosis (Zhao et al., 2018). In both *in vivo* and *in vitro* experiments, we found that a high-glucose environment significantly upregulated the expression of NLRP3 and pyroptosis pathway-related proteins, and CVB-D inhibited the expression of these proteins. Pyroptosis can deteriorate DNA breakage, so calcein/PI staining was used to identify pyroptosis and living cells. Experimental results showed that the number of PI staining positive cells in the CVB-D group was significantly lower than that in the HG group, showing that CVB-D prevented cardiomyocyte pyroptosis induced by HG. To further explore the relationship between CVB-D improving the PNRCM pyroptosis induced by HG and inhibition expression of NLRP3, we investigated the effect of pharmacological NLRP3 inhibitor (MCC950), agonist (BMS-986299), and NLRP3-shRNA lentivirus on the inhibition of PNRCM pyroptosis by CVB-D. There was no significant difference in the expression levels of NLRP3 and pyroptosis-related proteins between the two groups when the MCC950 or BMS-986299 was used in combination with CVB-D compared with the MCC950 or BMS-986299 alone under the condition of HG. The results suggested that CVB-D may ameliorate the HG-induced PNRCM injury by inhibiting cardiomyocyte pyroptosis *via* NLRP3 (Figure 9).

In summary, our experimental results are consistent with previous studies, and CVB-D has a significant improvement effect on DCM, which can reverse the cardiac dysfunction and pathological changes caused by DCM. The molecular mechanism may be involved that CVB-D inhibits cardiomyocyte pyroptosis *via* NLRP3 to improve DCM. The present results provide a novel idea for the clinical application of CVB-D.

## Data Availability

The original contributions presented in the study are included in the article/Supplementary Material; further inquiries can be directed to the corresponding authors.
